# Effectiveness of motivational interviewing in patients with dyslipidemia: a randomized cluster trial

**DOI:** 10.1186/s12875-015-0370-2

**Published:** 2015-10-24

**Authors:** Julia Bóveda-Fontán, Nieves Barragán-Brun, Manuel Campiñez-Navarro, Luís Ángel Pérula-de Torres, Josep M. Bosch-Fontcuberta, Remedios Martín-Álvarez, Juan Carlos Arbonies-Ortiz, Jesús Manuel Novo-Rodríguez, Margarita Criado-Larumbe, Jose Angel Fernández-García, Enrique Martín-Rioboó, Collaborative Group Estudio Dislip-EM (see list in Annex)

**Affiliations:** Primary Care Center Colmeiro, Vigo, Spain; CAP Vallcarca, Barcelona, Spain; Health Center Villarrubia (UGC Occidente), Teaching Unit of Family and Community Medicine Cordoba. Sanitary District Cordoba and Guadalquivir. Maimonides Institute for Biomedical Research of Cordoba (IMIBIC)/Reina Sofía University Hospital/University of Córdoba, Córdoba, Spain; Primary Care Center Encants (Maragall), Barcelona, Spain; Helth Center Errentería, Avda. Galzaraborda 67, Donostia, Spain; Centro de Saúde A Milagrosa, Lugo, Spain; UGC Fuensanta. Teaching Unit of Family and Community Medicine Cordoba. Maimónides Institute for Biomedical Research of Cordoba (IMIBIC)/Reina Sofía University Hospital/University of Cordoba, Córdoba, Spain; Unidad Docente de Medicina Familiar y Comunitaria de Córdoba. Distrito Sanitario Córdoba y Guadalquivir. C/Isla de Lanzarote, Córdoba, Spain; Maimónides Institute for Biomedical Research of Cordoba (IMIBIC)/Reina Sofia University Hospital/University of Córdoba, Córdoba, Spain

**Keywords:** Motivational interviewing, Behavior change counselling, Lifestyle change, Dyslipidemia, Cardiovascular risk factors

## Abstract

**Background:**

It is known that making people change their habits is challenging. It is crucial to identify the most effective approach that general practitioners (GPs) should use to help their patients change unhealthy habits. The objective this study was to assess the efficacy of a multifactorial intervention based on Motivational Interviewing performed by general practitioners to enhance lipid levels in patients with dyslipidemia, as compared to standard care.

**Methods:**

A multicenter, controlled, randomized, cluster, two-parallel arm trial with a 12-month follow-up conducted in 25 community health centers of the Spanish. 38 GPs and 227 primary care patients with uncontrolled dyslipidemia were included in the trial. GPs performed an intervention based either on Motivational Interviewing (MI) or standard practice. Lipid levels were measured, and the control degree was analyzed based on the criteria of clinical guidelines.

**Results:**

107 were assigned to the Experimental Group (EG) and 120 to the Control Group (CG). An overall improvement was achieved in total cholesterol levels (Mean Difference –MD- = −19.60; 95 % CI: −15.33 at −23.87 mg/dl; p < 0.001), LDL-cholesterol levels (MD = −13.78; 95 % CI: −9.77 at −17.79 mg/dl; p < 0.001) and triglycerides (MD = −19.14; CI 95 %: −11.29 at −26.99 mg/dl; p < 0.001). No differences were found between the two groups. However, when we assessed the degree of lipid control by combining cholesterol <200 mg/dl and LDL-cholesterol < 130 mg/dl parameters, it was observed that a higher percentage of patients achieved target figures in the EG versus CG (13.1 % vs. 5.0 %; adjusted OR = 5.77, 95 % CI: 1.67-19.91).

**Conclusion:**

A Motivational Interviewing-based approach conducted by Primary Care physicians aimed at patients with dyslipidemia, achieved a significant reduction in all lipid parameters, cardiovascular risk, weight reduction and the adherence to the Mediterranean diet, similar to that obtained with the usual intervention and superior in the proportion of patients achieving combined lipid control goals and the level of physical exercise.

**Trial registration:**

the trial is registered in ClinicalTrials.gov (NCT01282190; January 21, 2011).

**Electronic supplementary material:**

The online version of this article (doi:10.1186/s12875-015-0370-2) contains supplementary material, which is available to authorized users.

## Background

Cardiovascular diseases (CVD) are a major cause of death in developed countries [1]. CVD have a multifactorial origin, and dyslipidemia is one of the main risk factors for this type of disease. CVD prevention strategies are usually based on the identification and management of cardiovascular risk factors, many of which are related with lifestyle and habits. Making lifestyle changes by adopting healthy habits such as eating healthy foods and increasing physical activity are essential to the control of dyslipidemia and the prevention of CVD. In this sense, the European Guidelines on Cardiovascular Disease Prevention in Clinical Practice [2] highlight the role of GPs, who are considered key to initiating, coordinating and providing long-term follow-up for the prevention of CVD. GPs have an essential role in the identification of patients at risk for CVD.

It is known, however, that making people change their habits is challenging. It is crucial to identify the most effective approach that GPs should use to help their patients change unhealthy habits.

Motivational Interviewing, as defined [3] is a clinical approach aimed at increasing patients' inherent motivation for change by helping them explore and solve their ambivalences and resistance to change from a patient-centered approach. In the authors' words, "MI is a collaborative goal-oriented method of communication with particular attention to the language of change" [4]. It involves the conscious and disciplined use of specific communication principles and strategies based on the concept that clinical communication is a set of behaviors that can be adopted, observed and measured as any other clinical skills.

MI has been shown to be effective in a variety of contexts in helping patients change unhealthy habits [5–8]. However, more robust evidence is needed on its effectiveness in approaching health problems such as dyslipidemia in the primary care setting, since only a few studies are focused on changing this cardiovascular risk factor. It is noticeable the study by Mhurchú et al. [9] that included 121 patients with hyperlipidemia recruited in community health centers and referred to a hospital dietetic department, who were randomized to receive either standard or motivational dietary interventions designed by a dietitian. At three month follow-up, both groups had improved their dietary habits significantly and body mass indices, although lipid levels did not change. Other studies not specifically focused on patients with dyslipidemia did assess changes in blood lipid levels as a secondary outcome in an intervention aimed at modifying other cardiovascular risk factors [10–14]. However, only a few studies have been performed in a real clinical practice setting where MI was incorporated as a part of standard primary care.

All this said, we decided to perform a study to assess the effectiveness of using MI during patients' visits to the GP versus the standard communicational approach in favoring cardioprotective behaviors (heart protective diet, physical activity, weight loss) and improving the control of lipid levels, thus reducing cardiovascular risk.

## Methods

The protocol of this study has been published previously [15] (See Additional file 1).

### Study design

We carried out a multicenter, open, controlled, randomized, cluster, two-parallel arm trial (Experimental Group -EG- and Control Group -CG-) with a 12-month follow-up conducted in community health centers of the Spanish National Health System.

### Setting and participants

This trial was conducted using the cluster design, where two subpopulation levels were considered: [1] health professionals and [2] patients.

#### Health professionals

General practitioners at community health centers were recruited by convenience sampling. Inclusion criteria were: commitment to stay at their job at least one year, and signing an Confidentiality Agreement. GPs with previous training in MI were excluded.

A total of 54 GPs from 32 community health centers were finally included after excluding those who did not meet inclusion criteria or lost interest in the project.

#### Patients were recruited by the participating GPs

Inclusion criteria were being aged 40 to 75 years, having a de novo diagnosis of dyslipidemia based on the following simplified classification [16]: a) Defined hypercholesterolemia: total cholesterol >250 mg/dl and triglycerides <200 mg/dl; b) Hypertriglyceridemia: total cholesterol <200 mg/dl and triglycerides >200 mg/dl; or c) Mixed hyperlipidemia: total cholesterol >200 mg/dl and triglycerides >200 mg/dl.

Exclusion criteria were: Patients with conditions that may cause secondary dyslipidemia and need pharmacological therapy for their condition; patients with previous cardiovascular events or other chronic conditions as diabetes or severe chronic obstructive pulmonary disease, cancer, serious liver failure, chronic renal failure, alcohol or other substance abusers; patients who, because of their personal or labor characteristics, were unable to comply with the study procedures or to be subject to follow-up review; pregnant or nursing women; and patients prescribed hypolipidemic pharmacological treatment.

### Intervention

#### Intervention with health professionals

##### Intervention common to both groups

Before initiating patient recruitment, the participating health professionals attended a workshop on how to approach patients with dyslipidemia following a protocol specially designed for this trial and current clinical practice guidelines [17]. They also received training in consultation videotapes.

Finally, all participants were interviewed to assess their knowledge of patient interview and examine whether the groups were well-balanced.

In order to assess the GPs's baseline skills, before initiating the intervention with the patients physicians were video recorded as they consulted with two standardized patients with dyslipidemia trained in simulating office visits. Upon completion of the field work, physicians were video recorded again with two clinical cases similar to those seen at baseline and with the same actors. As a quality control measure and to assess physicians' adherence to the protocol, we recorded four real interviews between each participating physician and one of the patients recruited. All video recordings were scored using a Motivational Interviewing Assessment Scale (EVEM) [18]. Our research group previously demonstrated EVEM's reliability in assessing psychometric properties (intraclass correlation coefficient: >0.96; Cronbach's alpha: >0.95); as well as its validity and sensitivity to change. Change was assessed by determining differences in the scores obtained by the patients on the EVEM scale before and after the intervention. No differences were observed between the two groups at baseline, while differences were found -the higher the score, the more extensive the use of MI- both, after the training course for GPs (pre-training score = 23.63 vs. posttraining = 38.57; t = −4.549; p < 0.001), and in the evolution from the initial and the final visit (22.51 vs. 24.96, respectively; F = 3.039; p = 0.023-).

##### Intervention in the Experimental Group

Our research group designed a specific training program for the GPs included, which consisted of two parts: 1) A 16-hour training course delivered by an expert and focused on the eight basic MI tasks [19]. 2) After completion of the training program, two visits were recorded in which standardized patients simulated a situation similar to that of the visits previously recorded. Then, physicians attended an individual feedback session with an expert in MI. 3) Initial training was reinforced and maintained during field work through the following actions: a) each participant received "educational micropills" regularly via Internet and SMS messages; b) each participant was assigned a task and received feedback later; c) each participant attended group sessions to analyze their own visits with real patients using Problem Based Interviewing method [20].

##### Intervention in the control group

This group received no training in patient interviewing. The participants of this group were given strict instructions that his intervention should be the one normally developed in consultation with these patients.

#### Intervention with patients

Once physicians had been trained, the field work was initiated.

##### Patient recruitment

Patients were recruited by their general practitioner, who evaluated them in an initial visit to determine whether they met inclusion or exclusion criteria. The recruitment period was 18 months. Written consent was obtained from all patients.

##### Intervention with patients

The patients in the CG received standard care that consisted of providing advice on the necessity of changing unhealthy habits towards cardioprotective habits, according to clinical protocol recommendations. A MI-based approach was used in patients in the EG in combination with clinical protocol recommendations. Both interventions were performed by the patients' usual GPs and were integrated into standard primary care. The follow-up period was 12 months, with visits at baseline and at 2, 4, 8 and 12 months.

### Sample size

Basing on the results reported in previous studies [9, 21], assuming a 40 mg/dl variance in cholesterol levels, a SD = 15 mg/dl for total cholesterol, for an alpha error = 0.05 and a beta error = 15 %, 256 patients had to be recruited.

As this was a randomized cluster study, the "design effect" was taken into account during sample size calculations. Intra-cluster correlation coefficients (ICC) in cluster primary care trials are generally lower than 0.05 [22]. For a cluster size of 15, the ICC translated into a design effect of 1.7. Considering this value, the number of subjects to be recruited would be 218 for each group, whereas the number of GPs would be 48 to 50.

Although at the beginning a total of 91 GPs showed interest in participating in the trial, only 54 were finally included, since the other GPs did not meet the inclusion criteria or lost interest in the project. Thus, centered, blind, simple randomization was performed at a 1:1 ratio. For different reasons -either personal or professional-, 16 of the GPs included in the study left the trial before initiating patient recruitment. Therefore, the final number of GPs included in the study was 38, whereas the final number of patients recruited was 227. GPs recruited patients by consecutive sampling during their visits for whatever reason.

### Outcome measures

Variables collected during the visits were: age, sex, marital status, education level, social class, family status, family history of premature cardiovascular disease, comorbidity, current drugs consumption, snuff consumption (smoker, ex-smoker, non-smoker, number of cigarettes/day in case of smokers), and alcohol intake (basic units of alcohol/week), anthropometric data (weight, height, body mass index, waist circumference, blood pressure, heart rate), analytical data (total cholesterol, LDL-cholesterol, HDL-cholesterol, triglycerides, glucose, creatinine, uric acid, GOT/GPT/GGT, glomerular filtration rate), abnormal ECG, cardiovascular risk (SCORE, Framingham) [23, 24], diet (diet questionnaire Mediterranean) [25], physical activity (IPAQ questionnaire) [26], and adherence to medications (Haynes-Sackett and Morisky-Green tests) [27, 28].

### Measurement instruments

Data were recorded in two data collection logs (DCL), one for each group. In addition, GPs used a procedure manual including a clinical protocol for approaching patients with dyslipidemia [16].

### Coding procedure

Our research group revised the DCL and watched the video recordings to verify that data had been properly entered. When an error in data entry was detected, the study supervisor asked the corresponding researcher to correct the errors or deficiencies found.

### Statistical analysis

The statistical analysis included the following elements:

1. Descriptive analysis.
2. Baseline analysis: We compared sociodemographic and clinical characteristics of the EG with those of the CG.
3. Analysis of the effectiveness of the intervention: We also performed an intention-to-treat analysis including all the patients that received at least the first intervention.

The criteria used to consider good lipid control have been adjusted to the recommendations of clinical practice guidelines that were current at the time of the study design [16, 17] (total cholesterol <200 mg/dl and LDL-cholesterol <130 mg/dl or total cholesterol <200 mg/dl, LDL-cholesterol <130 mg/dl and triglycerides <200 mg/dl).

Finally, we assessed differences in primary and secondary outcomes between the two groups and among follow-up visits. Student's t-test was used for comparison of means of independent samples, whereas ANOVA, Chi-squared test or Fisher's exact or McNemar test were employed for repeated measures. When the outcomes did not follow a normal distribution (Shapiro-Wilk test), non-parametric tests were used, such as the Mann-Withney U test, Friedman test, or Kruskal–Wallis test (p ≤ 0.05). Multivariate statistics (Multiple Linear Regression or Unconditional Logistic Regression or Multilevel Logistic Regression, considering the doctor as a first level of analysis and the patient as a second level) is also applied. The independent variables considered in every model were sociodemographic, those clinical or epidemiological relevant, and the variable "intervention group" (Experimental vs. Control). For modeling we use the “Enter” method on SPSS. The qualitative variables were treated as categorical dummy variables. The goodness of fit of the logistic regression model was checked with the Hosmer-Lemeshow.

The study was approved by the Ethics Committee for Clinical Research of the Hospital Universitario Reina Sofía (Córdoba) and Ethics Committee for Clinical Research of Galicia.

## Results

### Description of the population sample and between-group baseline comparability

**Fig. 1:**
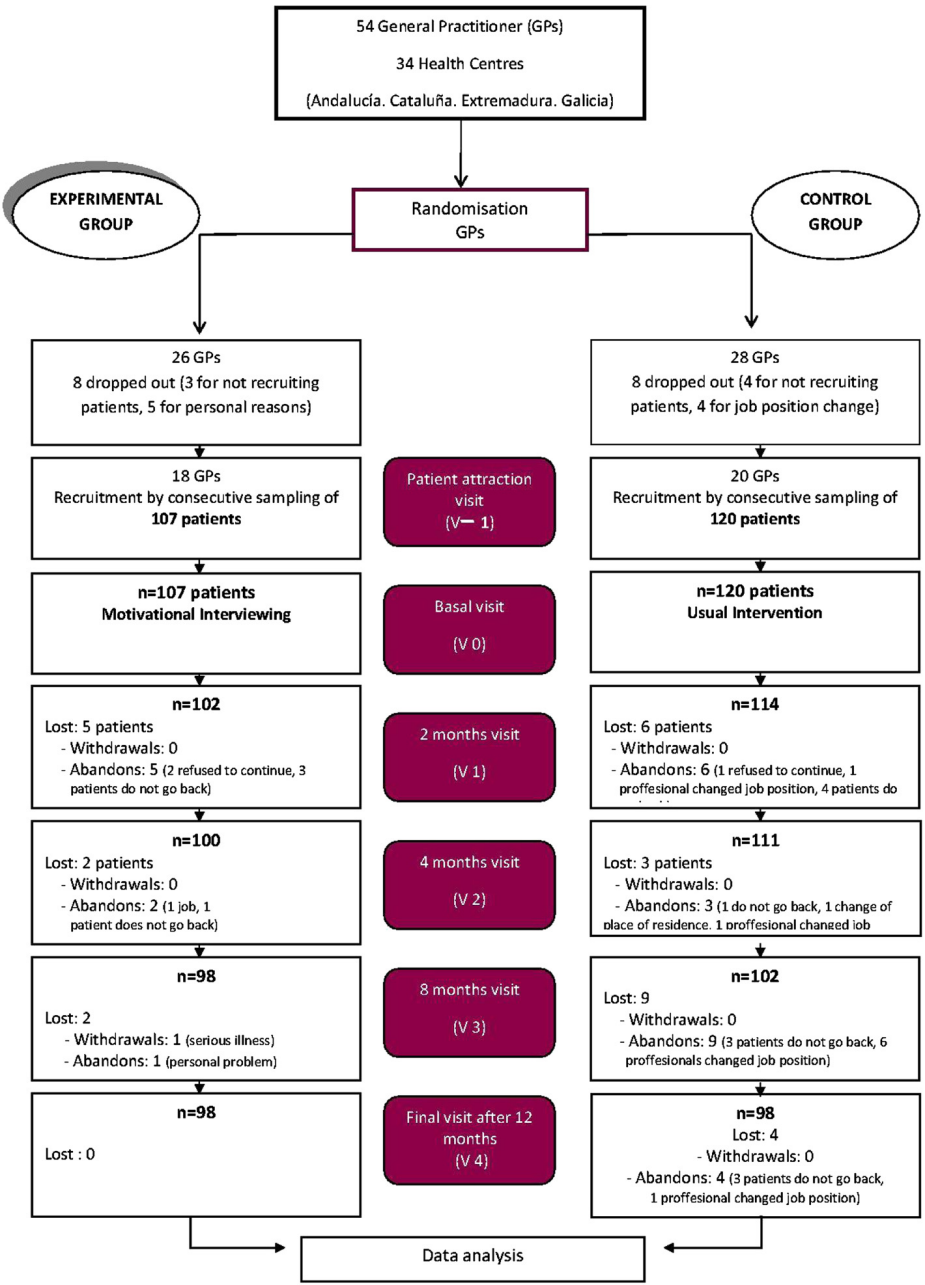
Flowchart of participants according to the CONSORT Group recommendations

The study included 227 patients who were recruited by 38 physicians from 25 community health centers in Spain. Eighteen physicians were assigned to the EG and 20 to the CG. The evolution and number of dropouts are detailed in the flowchart, following CONSORT Group recommendations (Fig. 1) [29]. Of the 227 patients included 107 were assigned to the EG and 120 to the CG. The mean number of recruited patients was 5.9 per GP for the EG (range: 1–10) and 6 for the CG (range: 2–10). A total of 196 patients −98 in each group- completed the follow-up period (lost-to follow up rate: 13.6 %).

Both groups were similar as to baseline characteristics (Tables 1 and 2). As shown on the flowchart, more women (62.1 %) than men were recruited to both groups; the average age was 53.7 years. As to marital status, a higher proportion of widowers and widows was observed in the EG.

**Table 1:** Sociodemographic characteristics and lifestyle of patients by baseline group (SD: Standard Deviation; HBP: High Blood Pressure; SDU: Standard Drinking Units; IPAQ: International Physical Activity Questionnaire)

VARIABLES	Experimental group n = 107	Control group n = 120	*P* value
AGE: Mean ± SD	52.83 ± 8.59	54.84 ± 8.53	0.079
SEX: n° (%)			
Women	63 (58.9)	78 (65.0)	0.343
Men	44 (41.1)	42 (35.0)	
MARITAL STATUS: No. (%)			
Single	3 (2.8)	8 (6.7)	0.015
Married/has a partner	91 (85.0)	92 (76.7)	
Separated	11 (10.3)	7 (5.8)	
Widowed	2 (1.9)	13 (10.8)	
EDUCATIONAL LEVEL: No. (%)			
No Education	9 (8.4)	12 (10.0)	0.523
Elementary Education	55 (51.4)	70 (58.3)	
Secondary Education	28 (26.2)	22 (18.3)	
Higher Education	15 (14.0)	16 (13.3)	
SOCIAL CLASS: No. (%)			
Class I (the highest)	9 (8.4)	10 (8.3)	0.230
II	6 (5.6)	4 (3.3)	
III	22 (20.6)	15 (12.5)	
IV	36 (33.6)	37 (30.8)	
V (the lowest)	34 (31.8)	54 (45.0)	
COMORBIDITY			
Arterial hipertension: No. (%)	25 (23.4)	30 (25.0)	0.774
Anxiety/Depression: No. (%)	28 (26.2)	33 (27.5)	
UNHEALTHY HABITS			
SMOKING: No. (%)			
Smoker	36 (33.6)	27 (22.5)	0.072
Former smoker	26 (24.3)	25 (20.8)	
Non-Smoker	45 (42.1)	68 (56.7)	
Years smoking (Mean ± SD)	26.94 ± 11.12	28.04 ± 12.20	0.727
ALCOHOL: No. (%)			
Non-drinker	59 (55.1)	75 (62.5)	0.385
Drinker	48 (44.9)	45 (37.5)	
SDU/week for smokers (Mean ± SD)	12.51 ± 12.55	8.11 ± 6.04	0.173
USE OF PHARMACOLOGICAL DRUGS: Mean ± SD			
Medicine	1.93 ± 1.94	2.55 ± 3.43	0.134
Number of pills/day	2.85 ± 2.16	3.51 ± 4.32	0.298
MEDITERRANEAN DIET:			
Questionnaire Score (Mean ± SD)	8.28 ± 2.23	8.32 ± 2.62	0.868
PHYSICAL ACTIVITY			
IPAQ: No. (%)			
Low or inactive	24 (22.4)	30 (25.0)	0.986
Moderate	44 (41.1)	52 (43.3)	
Intense	26 (24.3)	31 (25.8)	
Professional evaluation: No. (%)			
Sedentary	42 (41.2)	43 (36.4)	0.399
Active	60 (58.8)	75 (63.6)

**Table 2:** Patients' clinical and analytical data by baseline group (BP: Blood pressure, Heart Rate (bpm); BMI Body Mass Index, Adominal circumference (cm); AST: Aspartate Aminotransferase; ALT: alanine aminotransferase; GGT: Gamma Glutamyl Transpeptidase CKD-EPI: Chronic Kidney Disease Epidemiology Collaboration)

VARIABLES	Experimental group n = 107	Control group n = 120	*P* value
Systolic BP (mmHg): Mean ± SD	129.26 ± 14.54	130.17 ± 15.67	0.595
Diastolic BP (mmHg): Mean ± SD	79.04 ± 9.11	77.90 ± 9.49	0.228
Heart rate (bpm) (mmHg): Mean ± SD	74.08 ± 10.06	74.18 ± 9.59	0.927
BMI: Mean ± SD	28.47 ± 3.90	28.41 ± 4.47	0.670
Waist Circumference (cm): Mean ± SD	94.47 ± 12.00	95.15 ± 11.51	0.744
Family history of early CVD: n (%)	24 (22.4)	16 (13.3)	0.073
ECG: n (%)			0.466
Normal	95 (88.8)	105 (87.5)	
Alterations	7 (6.5)	5 (4.2)	
Unknown	5 (4.7)	10 (8.3)	
ANALYTICAL DATA: Mean ± SD			
Total Cholesterol (mg/dl)	263.44 ± 28.26	259.26 ± 26.09	0.098
LDL Cholesterol (mg/dl)	171.89 ± 27.28	171.10 ± 28.56	0.734
HDL Cholesterol (mg/dl)	57.03 ± 17.63	55.55 ± 14.53	0.508
Triglycerides (mg/dl)	170.64 ± 87.93	170.30 ± 106.05	0.516
Creatinine (mg/dl)	0.83 ± 0.17	0.79 ± 0.19	0.039
Glucose (mg/dl)	95.65 ± 10.68	95.76 ± 17.19	0.463
Uric Acid (mg/dl)	4.86 ± 1.52	4.90 ± 1.56	0.883
GOT (UI/l)	22.60 ± 7.96	24.26 ± 11.67	0.658
GPT (UI/l)	25.65 ± 12.0	26.29 ± 16.90	0.693
GGT (UI/l)	38.16 ± 35.47	36.14 ± 29.71	0.966
CKD-EPI (ml/min/1.73 m^2^)	91.06 ± 12.92	92.98 ± 15.06	0.135
VASCULAR RISK: Mean ± SD			
SCORE (%)	1.32 ± 1.50	1.54 ± 1.72	0.346
REGICOR (%)	4.48 ± 2.61	4.47 ± 2.50	0.693
FRAMINGHAM (%)	9.52 ± 6.05	9.98 ± 6.37	0.447

### Results by outcome

#### Lipid parameters (Figs. 2, 3 and 4)

The mean level of cholesterol dropped significantly in the two groups during the follow-up period (mean difference -MD- = −19.60; 95 % 95 % CI: −15.33 to −23.87 mg/dl; Friedman test = 91.756; p < 0.001), with no differences between the two groups (F = 0.021; p = 0.996). Multivariate analysis showed that the predictors of total cholesterol levels as measured at the end of the study were (F = 4.765; p < 0.001; R^2^ = 0.464): the participating physician (p = 0.050), adherence to Mediterranean Diet (p < 0.001), baseline cholesterol level (p < 0.001) and use of statins (p < 0.001).

**Fig. 2:**
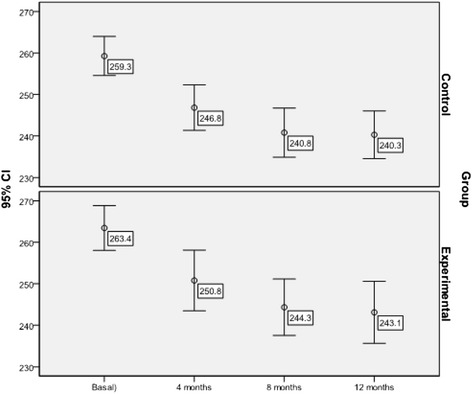
Evolution of mean total cholesterol levels throughout the study by group (mg/dl)

**Fig. 3:**
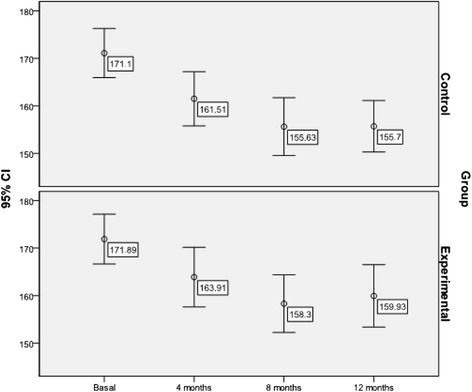
Evolution of mean LDL- cholesterol levels throughout the study by group (mg/dl)

**Fig. 4:**
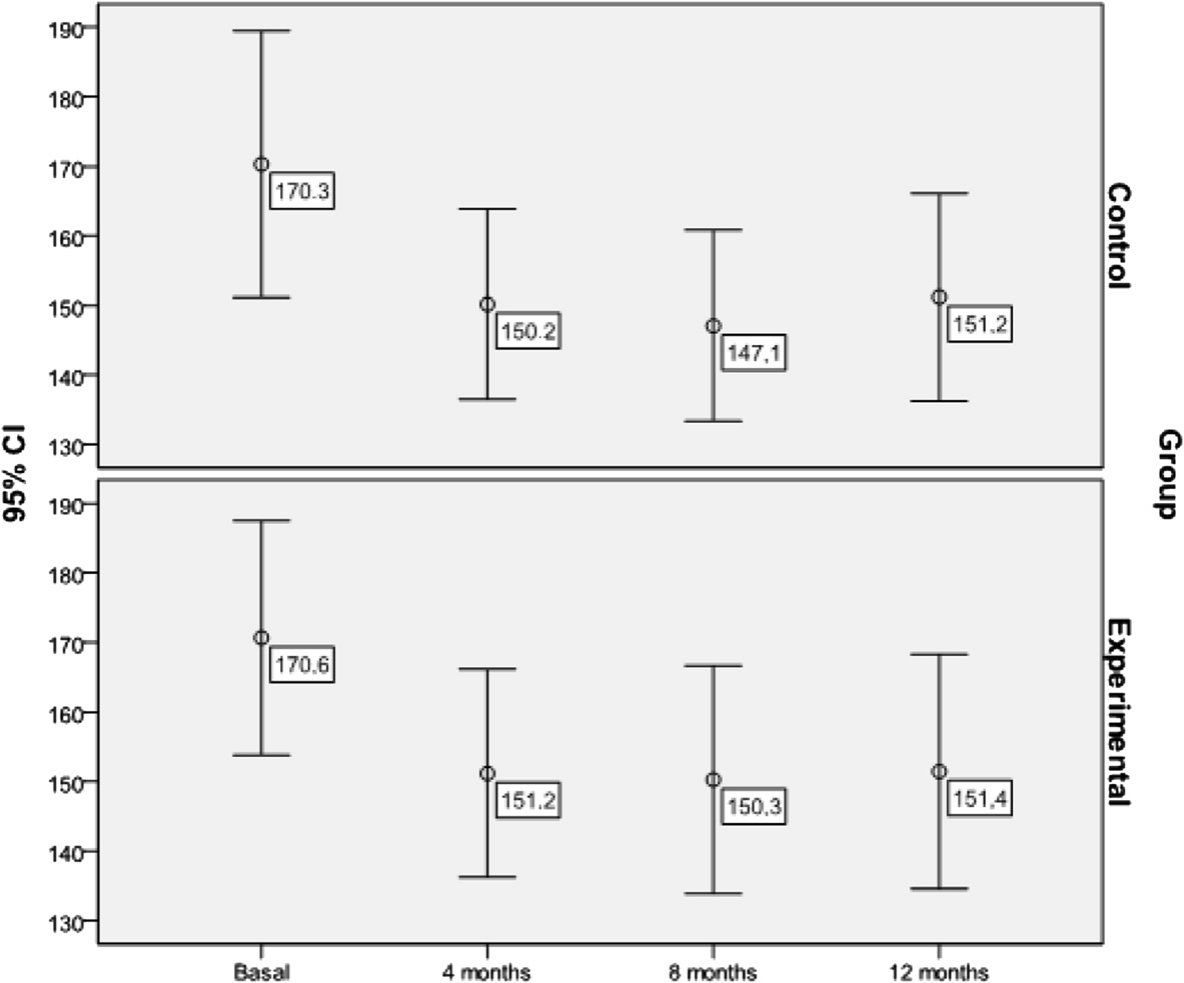
Evolution of mean total triglyceride levels throughout the study by group (mg/dl)

Similarly, a significant reduction in mean LDL-cholesterol levels was achieved in the total sample (MD = −13.78; 95 % CI: −9.77 to −17.79 mg/dl; Friedman test = 58.856; p < 0.001), with no significant differences between the groups (F = 0.067; p = 0.977). Predictors of LDL-cholesterol levels at the end of the study were (F = 2.643; p = 0.001; R^2^ = 0.392): Mediterranean Diet (p = 0.011), baseline LDL-cholesterol level (p < 0.001) and use of statins (p = 0.004).

The mean triglyceride level was reduced significantly in both groups (MD = −19.14; 95 % CI: −11.29 to −26.99 mg/dl; Friedman test = 23.390; p < 0.001), with no differences between the two groups (F = 0.216; p = 0.886). The multivariate model (F = 2.643; p = 0.001; R^2^ = 0.596) showed that the only predictor of triglyceride levels measured at the end of the study was baseline triglyceride levels (p < 0.001).

Conversely, no significant differences were observed in HDL-cholesterol levels after the intervention when assessed globally (MD = 0.28; 95 % CI: −2.26 to 1.69 mg/dl; Friedman test = 3.591; p = 0.309). As it occurred with the other lipid parameters, no significant differences were found in HDL-cholesterol levels between the groups (F = 0.048; p = 0.826).

**Fig. 5:**
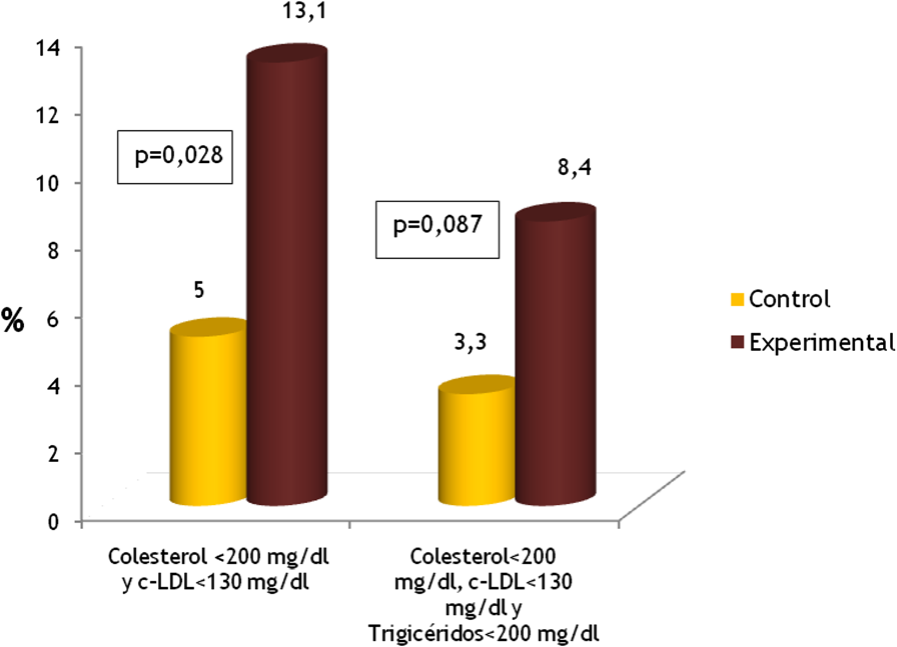
Patients with lipid control in the final visit depending on the group

When calculating the control degree by groups according to the therapeutic objectives achieved at the end of the study with the LDL-cholesterol < 130 mg/dl criterion, no statistically significant differences were noted. On the other hand, when we analyzed the combined cholesterol < 200 mg/dl and LDL-cholesterol < 130 mg/dl (Fig. 5) lipid parameters, a higher percentage of patients reached target figures in the EG against CG (13 1 %. vs. 5 %; Chi-square = 4.601; p = 0.028). Moreover, when we introduced the triglyceride parameter, i.e. cholesterol < 200 mg/dl, LDL-cholesterol < 130 mg/dl and triglycerides < 200 mg/dl, differences were seen also for EG (8.4 % vs. 3.3 %), even if they were of little significance (Chi-square = 2.744; p = 0.087). Table 3 shows the multivariate analysis which shows that the predictors of the control degree based on the combined cholesterol < 200 mg/dl and LDL-cholesterol < 130 mg/dl endpoint were the group (OR = 5.77), the physician (OR = 1.07) and the use of statins (OR = 7.19). The multivariate analysis on Table 4 shows that the predictors of the control degree based on the combined cholesterol < 200 mg/dl, LDL-cholesterol < 130 mg/dl and triglycerides < 200 mg/dl endpoint were the group (OR = 8.41), use of statins (OR = 20.70) and the Mediterranean diet score at the final visit (OR = 1.63).

**Table 3:** Multivariate analysis (multiple logistic regression) with independent variables considered taking lipid control level based on the total <200 mg/dl and c-LDL < 130 mg/dl colesterol (*n* = 227; OR = Odss Ratio; 95 % CI: 95 % Confidence Interval; Omnibus test = 35.063; *p* = 0.038; Hosmer-Lemeshow test = 8.312; *p* = 0.319)

Independent variables	*p*	OR	95 % CI OR	
			Lower	Upper
Group (Experimental/Control)	0.005	5.77	1.67	19.91
Doctor	0.034	1.07	1.00	1.13
Age	0.252	0.95	0.88	1.03
Sex (Male *vs* Female)	0.587	1.46	0.36	5.87
Instruction level	0.731			
Instruction level (Uneducated vs Higher)	0.998	0.00	0.00	
Instruction level (Primary vs Higher)	0.887	1.19	0.09	14.72
Instruction level (Secondary vs Higher)	0.554	0.47	0.04	5.55
Profession (social class)	0.893			
Social class I vs V	0.574	0.40	0.01	9.34
Social class II vs V	0.806	0.66	0.02	18.09
Social class III vs V	0.916	0.90	0.13	6.04
Social class IV vs V	0.377	0.49	0.10	2.34
Family Situation (live vs accompanied only)	0.857	0.86	0.16	4.40
Anxiety/Depression	0.801	1.19	0.29	4.80
Smoking final visit	0.456	0.56	0.12	2.52
Alcohol consumption final visit	0.346	0.54	0.15	1.94
Delivery of written information	0.989	1.00	0.29	3.44
BMI final	0.221	1.08	0.95	1.23
Mediterranean diet final visit	0.312	1.15	0.87	1.53
Changes in diet at the end	0.834	1.15	0.31	4.27
Changes exercise final visit	0.066	0.27	0.06	1.08
IPAQ medium/high final visit	0.658	0.70	0.15	3.26
Statins use	0.004	7.19	1.85	27.93
Constant	0.425

**Table 4:** Multivariate analysis (multiple logistic regression) with independent variables considered taking lipid control levels (cholesterol <200 mg/dl, c-LDL < 130 mg/dl and triglycerides <200 mg/dl) as the dependent variable (OR = Odds Ratio; 95 % CI = 95 % confidence interval; Omnibus test = 38.597; *p* = 0.016; Hosmer-Lemeshow test = 8.262; *p* = 0.408)

	*p*	OR	95 % CI OR	
Independent variables			Lower	Upper
Group (Experimental/Control)	0.020	8.41	1.40	50.459
Doctor	0.302	1.04	0.96	1.137
Age	0.145	0.91	0.82	1.030
Sex (Male *vs* Female)	0.172	3.91	0.55	27.760
Instruction level	0.737			
Instruction level (Uneducated vs Higher)	0.998	0.00	0.00	.
Instruction level (Primary vs Higher)	0.268	11.82	0.15	933.239
Instruction level (Secondary vs Higher)	0.362	7.58	0.09	589.011
Profession (social class)	0.799			
Social class I vs V	0.469	5.85	0.04	701.661
Social class II vs V	0.341	9.92	0.08	1122.703
Social class III vs V	0.689	0.59	0.04	7.812
Social class IV vs V	0.687	0.64	0.07	5.393
Family Situation (live vs accompanied only)	0.720	0.56	0.02	12.659
Anxiety/Depression	0.661	0.67	0.11	3.946
Smoking final visit	0.772	0.74	0.09	5.666
Alcohol consumption final visit	0.060	0.16	0.02	1.076
Delivery of written information	0.882	1.13	0.22	5.694
BMI final	0.680	1.04	0.86	1.250
Mediterranean diet final visit	0.028	1.63	1.05	2.550
Changes in diet at the end	0.429	0.46	0.07	3.098
Changes exercise final visit	0.210	0.29	0.04	1.994
IPAQ medium/high final visit	0.501	2.56	0.16	39.708
Statins use	0.001	20.70	3.40	125.838
Constant	0.408			

#### Other outcomes measured

As many as 80.4 % of the patients assigned to the EG and 79.2 % of patients in the CG had overweight or obesity. The proportion of obese patients was lowered by 8.4 % in the EG vs. 6.7 % in the CG, which means a 1.7 % difference between both groups (the reduction was more prominent in the EG) (McNemar = 13.899; p = 0.001). When considering only patients with overweight or obesity, we found that the mean weight dropped from 78.76 ± 11.27 kg at baseline to 76.92 ± 12.13 kg at the end of the study (MD = −1.77 kg; 95 % 95 % CI: −0.91 to −2.64 kg; Friedman = 47.599; p < 0.001). However, differences between the two groups were not significant (F = 1.258; p = 0.285). A reduction in body mass index (BMI) from 29.79 ± 3.53 kg/m^2^ to 29.17 ± 3.54 kg/m^2^ was also observed in these patients. Although this reduction is not statistically significant when the two groups are compared (F = 0.567; p = 0.452), if we consider the total sample such difference becomes significant (MD = −0.61 kg/m^2^; 95 % CI: −0.34 to −0.88 kg/m^2^; Friedman = 59.050; p < 0.001). The MD in the EG was also significant = −0.43 kg/m^2^; 95 % CI: −0.08 to −0.79 kg/m^2^).

As to waist circumference, we observed a significant reduction in the total sample (MD = −0.100 to −1.607 cm; Friedman = 47.086; p < 0.001), with no differences between groups (F = 0.927; p = 0.449). A significant reduction in waist circumference was observed in the total sample of patients with obesity or overweight (MD = −079 cm; 95 % CI: −0.287 to −1.746 cm; Friedman test = 34.272; p < 0.001), from 98.20 ± 9.67 cm to 97.47 ± 8.90 cm at the end of the study, with no significant differences between groups (F = 0.545; p = 0.703).

The level of physical activity was measured using the International Physical Activity Questionnaire (IPAQ Questionnaire) which showed an increase of physical activity in both groups (p = 0.030), although it was significantly more prominent in the EG (Chi-squared = 23.3; p < 0.01). As many as 96.6 % of patients in the EG reported a moderate to high level of physical activity at the end of the study.

The questionnaire on adherence to the Mediterranean diet yielded a mean score of 8.30 ± 2.43 at baseline, and a final score of 9.41 ± 2.47 (MD = 1.11; 95 % CI: 1.42-7.29; Friedman test = 44.366; p < 0.001) after a one-year follow-up period. In the final visit, a positive change was observed in the two groups, with no significant differences between them (95 % CI of MD: −0.626 to 0.582).

The average points obtained on the SCORE risk charts dropped significantly for the total sample (MD = −0.17 %; 95 % CI: −0.07 to −0.27; Friedman test = 20.596; p < 0.001), and Framingham (MD: −1.22 %; 95 % CI: −0.81 to −1.63; Friedman test = 34.794; p < 0.001), with no statistically significant differences between the EG and the CG.

The analysis of consumption of snuff reflects a significant reduction in the number of smokers from the first to the last visit in the total sample: 37.0 % reduction in the CG (McNemar; p < 0.001) and 33.0 % in EG (McNemar; p = 0.012), with no differences between groups. A significant decrease in average number of cigarettes/day in smokers (95 % CI = −3.32 to −7.94; mean difference = −5.66; Friedman test = 46.732; p < 0.001) was evident, without significant differences between groups (F = 0.103; p = 0.749).

The average alcohol consumption at baseline, among subjects who reported drinking it regularly was 8.11 ± 6.04 U/week in CG and 12.51 ± 12.55 U/week at GE. At the final visit the average consumption dropped to 7.89 ± 7.46 in the CG and 10.62 ± 9.84 in the GE, the global average decline was 8.92 U/week (95 % CI: −6.84 to −11.01; t = 8.502; p < 0.001). Only 7 of the 227 patients reported consuming alcohol risk at baseline (6 at the GE and 1 in the GC). Of these, only three remained hazardous drinking at the final visit (2 in the GE and 1 in the GC).

No significant changes were observed in blood glucose levels or other biochemical parameters analyzed.

Finally, a higher proportion of patients in the CG were prescribed statins at the end of the study (19.2 %) as compared to patients in the EG (9.3 %); − Chi-squared = 5.042; p = 0.025-. Therapeutic adherence to the end of the follow-up period was 88.5 %, with no significant differences between groups (n = 26; Chi-square = 2.052; p = 0.152).

## Discussion and conclusion

### Discussion

#### Main findings

We observed a global improvement in the lipid parameters analyzed, except for HDL-cholesterol. Reductions were significant both in the patients who received standard care and in the patients who received the motivational interviewing-based intervention. No statistically significant differences were observed.

However, when we analyzed the percentage of patients who had achieved target figures at the end of the study, we observed a significantly higher proportion of patients treated in the GE, both when considering the combination of cholesterol < 200 mg/dl and LDL-cholesterol < 130 mg/dl, and the combination of cholesterol < 200 mg/dl, LDL-cholesterol < 130 mg/dl and triglycerides < 200 mg/dl as well, and adjusting these response variables with other presumed predictors in a multivariate analysis. This disparity or alleged discrepancy as to the outcomes can be explained from a statistical point of view, because when data are treated as quantitative variables, the used comparison parameter has been the arithmetic mean and other descriptive measures have been ignored, such as the bias or asymmetry of the distributions, whereas when the used end-point is qualitative, and also in combination (i.e. using as criteria some specific cut points to qualify patients as controlled or not controlled), differences come to the fore which were not appreciated as quantitative measures.

It is to be noticed that total cholesterol levels were dramatically reduced and maintained for a year.

No significant correlations were observed between changes in lipid parameters and independent variables such as sociodemographic or unhealthy habits of the patient.

No associations were found either between self-reported level of physical activity in the final visit and changes in lipid parameters, despite the increase in physical activity.

An association was found between the use of statins and a higher score on the Mediterranean Diet questionnaire and total cholesterol and LDL-cholesterol levels. However, no association was found between the use of statins and triglyceride levels.

We observed a positive correlation among the three parameters at baseline and at the end of the study.

A correlation was also found between the participating physician and total cholesterol levels -regardless of the type of intervention-, which led us think that other behavior al factors and professional skills may have an impact on the outcomes.

#### Interpretation of findings and comparison with existing literature

The changes observed in serum cholesterol levels were more prominent than those reported in previous studies. In a review on the effectiveness of individual interventions in primary care settings aimed at changing life habits to reduce cardiovascular risk reported a mean reduction of 12.76 mg/dl in total cholesterol levels [30]. It is to be noticed that this review included trials that involved a pharmacological intervention, provided that it was not the primary outcome. The same author considered that reductions of 19.3 mg/dl were clinically relevant.

According to Gotto & Pownall [31], a 10 % reduction in total cholesterol leads to a mean 8 % reduction in all causes of mortality. Other data indicates that a slight reduction (3 %) of total cholesterol results in a 15 % reduction of cardiovascular events [32]. Conversely a 40 mg/dl reduction of LDL-cholesterol levels is associated with a 22 % reduction of cardiovascular morbidity and mortality [33].

Considering these data and taking into account that the study population had no previous history of cardiovascular events, we consider that the changes achieved in our study are clinically very relevant as to their potential for reducing the incidence of cardiovascular disease. In addition, cholesterol levels at baseline were only slightly high, which made it more difficult to achieve significant variations.

The physicians in the CG prescribed statins for the treatment of dyslipidemia more frequently than those in the EG. This means that there was disparity between the two groups as to the use of pharmacological drugs, which may be a confounding factor in the interpretation of the results obtained.

This is noticeable, given that both groups had a similar health state at baseline. Although similar reductions were achieved in both groups, more pharmacological drugs of proven efficacy in reducing lipid levels were prescribed to the CG.

When pharmacological prescriptions are isolated, a mean reduction of 17.71 mg/dl in total cholesterol levels is obtained for the EG vs. 12.53 mg/dl for the CG. Therefore, the reduction of total cholesterol levels achieved by the GPs using motivational interviewing (6.72 %) without any pharmacological treatment were substantially greater as compared to those obtained in the CG (4.83 %).

Although this difference was not statistically significant, it is undoubtedly of interest and relevant to the final outcomes as the multivariate analysis shows. Thus, the multivariate analysis confirmed that the use of statins is one of the main predictors of changes in lipid levels.

This finding suggests that the physicians who used MI tended to prescribe statins less frequently and only used them when it was strongly recommended by clinical guidelines recommendations. This means that the use of MI may result in a lower use of pharmacological drugs, which would potentially reduce associated health care costs and the risk of statin-related side effects.

The existing literature reports that both, dietary interventions alone and multifactorial interventions reduce cholesterol levels only slightly (3-5 %) [34].

A Cochrane review [35], was conducted to assess the effectiveness of dietary advice in improving cardiovascular risk in healthy adults, reported a mean reduction of cholesterol levels of 6.19 mg/dl in 3 to 24-month follow-up periods. According to Cochrane, the outcomes improved when the dietary intervention was performed by a dietitian rather than by a physician.

However, some studies have demonstrated that patients could not maintain LDL-cholesterol levels low in the long term when the intervention was performed by a dietitian [36].

In our study, the mean weight reduction in patients with obesity or overweight was 1.78 kg (2.3 % of baseline weight).

It is essential to maintain weight loss for one year, since it is the main problem reported by most intervention studies on weight loss [37]. Some authors consider a ≥1 kg weight loss clinically relevant [30]. Other studies reporting greater weight losses combined dietary and exercise interventions with a behavior approach. However, exercise programs were difficult to comply with [38]. Other studies report significantly greater weight losses achieved using a motivational intervention. However, the intensity of the intervention was higher in the motivational group than in the control group, and weight was measured only after six months of intervention [39].

A meta-analysis assessing the effectiveness of MI in patients with overweight or obesity [40] concluded that MI contributed significantly to weight loss as compared to controls and highlighted the fact that in several studies both, the intervention and the control group achieved significant weight losses. In addition, those studies reporting weight loss as the primary endpoint showed better outcomes than those focused on changing life habits in general [40].

Dietary habits improved in our study population, with a mean final score exceeding the threshold indicating good adherence [25].

It was also noticeable the increase in physical activity in the EG, which obtained significantly higher scores on the IPAQ questionnaire than the CG. This is supported by the high number of patients in the EG reporting to have moderate to high physical activity in the end of the study, whereas only a small proportion of patients in this group (6.3 %) reported that they kept being sedentary.

Although multivariate analysis indicated that there was no correlation between the high level of physical activity achieved and the reduction in lipid parameters, it is undeniable that the increase in physical activity is very relevant. In fact, there is solid evidence that regular physical activity is effective in primary and secondary prevention of different chronic diseases [41]. The relative risk of developing ischemic heart disease associated with sedentarism ranges between 1.5 and 2.4 [42].

Also, there is evidence that there is a progressive linear relation between the level of physical activity and health status; thus, people who are more active physically are at a lower risk of developing heart disease [43]. This finding is even more interesting if we consider the moderate efficacy of the interventions at increasing physical activity reported in the literature aimed [44].

Other studies performed in the primary care setting have demonstrated the efficacy of MI-based interventions in reducing sedentarism. However, these interventions designed by health professionals are generally more intensive and occasionally include support personnel such as experts on physical activity, who complement the intervention through personal interviews, phone calls or even mailed material [10, 11, 45]. Even in more intensive interventions, improvements have been reported in the long term for the two groups [46].

The existing literature indicates that lifestyle interventions in primary care aimed at patients at low CVR cardiovascular risk are barely effective or, at least, there is no sufficient evidence on their effectiveness when a multifactorial approach is used [47, 48]. This is the case of comprehensive interventions aimed at improving dietary habits, physical activity and weight in patients with lipid disorders. Some studies report that MI is more effective in patients at a higher baseline risk of CVD [49]. The studies using MI in patients with lipid disorders report a variety of outcomes.

Thus, Woollard [14] did not found any significant differences in the lipid profile, dietary habits or weight of patients after an intervention performed by nurses trained in MI, as compared to standard advice delivered by GPs to patients with high cardiovascular risk. Woollard reported a 3 % reduction of total cholesterol in the EG vs. a 2 % reduction in the CG at 12 months.

Elley [10] and Lawton [11] performed two primary care interventions aimed at increasing PA in a sedentary population using a motivational approach. However, they did not achieve to increase physical activity, weight loss or reduce blood pressure or lipid levels, which were secondary outcomes.

We did not find any other trial where GPs highly trained in MI have implemented an efficient intervention where biological parameters associated with health habits were measured for a year. We only found a similar study [50] that showed that training primary care professionals in Behavior Change Counseling was not more effective in achieving changes in life habits or in biochemical or anthropometric parameters, as compared to standard care.

Some studies have reported positive effects of MI on biological parameters. However, these studies have methodological limitations.

Kreman [51] reports a significant reduction of total and LDL-cholesterol levels in patients with dyslipidemia using a single motivational intervention through the telephone. But the sample size was very small and the study period very short.

There are a large number of publications on MI interventions performed in primary care; however, they were not implemented by experts on MI or were integrated into regular practice. Thus, the study by Hardcastle [49] in the United Kingdom was performed in a community health center by experts in PA and dietitians rather than by GPs.

Another important finding of our study was the difference found in the overall percentage of dropouts during the follow-up period. There were more dropouts in the CG (18.4 %) than in the EG (8.5 %), which means that adherence was significantly higher in the EG. The higher adherence in the EG might be due to the effect of MI, which favors patient's engagement (one of the four pillars of MI) and the quality of patient-physician communication.

#### Strengths and limitations of this study

Although this trial was implemented in experimental conditions, they were very similar to conditions in regular primary care. This gives greater external validity to the study, since conditioning factors such as care overload and time constraints were considered.

An intervention based on the patient-doctor relationship should be assessed at two levels: the acquisition of skills by health professionals and the intended behavior change in patients. All authors highlight the importance of considering aspects such as professionals skills, recruitment methods and adherence to intervention protocols [52].

Madson & Campbell [53] emphasized the need for effective objective observation tools for assessing MI reliability and quality.

Internal validity was achieved by implementing comprehensive control measures during the study, from the selection of the study design (randomization in groups), to the use of an educational program for physicians based on the eight MI skills. In addition, we assessed participants' adherence to the intervention protocol and verified its reliability; implementation differences between the two arms of the study were assessed using a measure tool previously validated (EVEM) [18].

It is understood that physicians may have considerable influence on patients' health-related behaviors [54]. On the other hand, a limitation of this study is the fact that the physicians who agreed to participate in such a complex trial may have been those who were more skilled and motivated to improve their communication skills and patient-centered practice, which would result in self-selection bias. Thus, the participating physicians might be more skilled than the average physician [55].

But the main limitation of this study is the rate of participation among physicians, which was lower than the rate initially achieved (38 of the initial 50). This would lead to the self-selection bias mentioned above and to randomized errors, since we worked with a statistical power which was less than desirable (type II error).

The low rate of participation among physicians hindered patient recruitment, which led to a final sample smaller than expected. This sample size allowed us to detect statistically significant differences, assuming a mean total cholesterol level of 235 mg/dl in the EG and 250 mg/dl in the CG, with a standard deviation of 24.0 mg/dl for an alpha risk of 5 % and a beta risk of 5 %, and considering the estimations described in section Methods.

A potential source of variation in MI effectiveness is the nature of the sample treated. The characteristics of patients may moderate the effectiveness of MI, since those initiating the treatment who already are ready for change may benefit less from MI because their ambivalence is already overcome [4].

Often, patients who decide to participate in a trial already have the motivation for change before the intervention. The percentage of drop-outs was higher in the CG than in the EG. This means that adherence was higher in the EG, which might lead to differential selection bias and affect the outcomes, since non-respondents are usually poorly controlled and do not follow recommendations.

On the other hand, this study might be subject to attentional bias -Hawthorne effect [55]- by which just the fact of being included in a study -and even before receiving the intervention- the subject may present behavioral changes. This might explain the improvements also observed in the CG, which in this case might affect both, professionals and patients. It is difficult to avoid this bias, since it is inherent to most trials, especially to experimental trials.

A common concern among primary care professionals are time constraints on interventions. However, the meta-analysis by Van Buskirk [56] showed that the total time devoted to patient-physician communication was not relevant to effect size. In our study we could not assess differences in the duration of patient-physician interviews due to the low number of GPs who recorded correctly this data.

### Conclusion

Both MI and Standard Care significantly reduce total cholesterol, LDL-cholesterol and triglyceride levels after a one-year follow-up program. The same thing happens with the reduction of the CVD risk, the adherence to the Mediterranean diet and the reduction of the corporal weight.

MI is superior than Standard Care in the proportion of patients achieving objectives combined lipid control and manages patients to increase their level of physical exercise in a greater degree than the usual approach.

Therefore, the EM can have an effect in patients with dyslipidemia in primary care but it is necessary to continue with quality studies that corroborate it.

MI is a useful clinical method in primary care that can be learned and used by health professionals to achieve changes in lifestyle of patients. The utility of MI in primary care stems from the possibility of combining it with other educational (advice, counseling…) and clinical-pharmacological interventions.

## Additional file

Additional file 1:
**CONSORT 2010 checklist of information to include when reporting a cluster randomised trial.** (DOC 131 kb)

## Abbreviations

BMI: Body mass index
CG: Control group
CVD: Cardiovascular
DCL: Data collection logs
COPD: Chronic obstructive pulmonary disease
EG: Experimental group
EVEM: Motivational Interviewing Assessment Scale
HDL-cholesterol: High-density lipoprotein-cholesterol
ICC: Intra-cluster correlation coefficients
IPAQ: International physical activity questionnaire
LDL-cholesterol: Low-density lipoprotein-cholesterol
MD: Mean difference
MI: Motivational Interviewing
OR: Odds ratio
SD: Standard deviation
